# Proteolytic activities of extracellular vesicles attenuate A-synuclein aggregation

**DOI:** 10.1038/s41531-025-01122-9

**Published:** 2025-09-29

**Authors:** Kostas Vekrellis, Agaristi Lamprokostopoulou, Katerina Melachroinou, Marianna Kokoli, Eleni Zingkou, Marina Skarveli, Asimina Kolianou, Martina Samiotaki, Georgia Sotiropoulou

**Affiliations:** 1 Center for Basic Research, Biomedical Research Foundation Academy of Athens, Athens, Greece; 2 Department of Pharmacy, School of Health Sciences, University of Patras, Rion-Patras, Greece; 3 BSRC Fleming, Vari, Greece

**Keywords:** Biochemistry, Cell biology, Neuroscience, Pathogenesis

## Abstract

Extracellular vesicles (EVs) are nano-sized lipid vesicles released into the extracellular space. We investigated the role of mouse brain-derived EVs in α-synuclein (α-syn) degradation and pathology transmission. Using sucrose gradient isolation and biochemical characterization, we found that EVs harbor active proteases that cleave both monomeric α-syn and pre-formed fibrils (PFFs). Protease activity and inhibitor profiling identified cathepsins B and S as key enzymes mediating this cleavage. EV-mediated proteolysis reduced the seeding capacity of α-syn PFFs in vitro and in vivo, whereas protease inhibition enhanced aggregation. Proteomic analysis revealed a restricted protease repertoire within EV cargo. Our findings suggest that EVs regulate extracellular α-syn levels via proteolysis, thereby modulating its prion-like spreading potential. We suggest that EVs represent a novel post-translational mechanism to regulate the levels of extracellular α-syn and may thus affect the spreading of α-syn pathology. Targeting this proteolytic capacity may offer new therapeutic interventions for mitigating synucleinopathies.

## Introduction

Considering the α-synuclein (α-syn) burden hypothesis prevailing the pathogenesis of Parkinsons’s disease (PD) and related synucleinopathies, it is important to understand not only the mechanisms by which the α-syn protein is produced but also the pathways by which it is cleared. It is possible that dysfunction in clearing mechanisms may contribute to the development and progression of PD. Importantly, secreted α-syn has been implicated in the transmission of pathology in PD brain^1^, in a manner that resembles prion protein transmission^2^. Extracellular α-syn levels are determined by both the rate of its release from neuronal cells and the rate of its elimination via various clearance pathways^3,4^. Although the exact mechanisms that account for the clearance of extracellular α-syn species are not fully elucidated, there is accumulating evidence that these mechanisms include proteolysis by extracellular proteases, as well cell-mediated uptake and intracellular degradation. In this respect, we have previously shown that α-syn species can be secreted in association with extracellular nano-sized vesicles of endosomal origin termed extracellular vesicles (EVs)^5^. Nevertheless, the possible role(s) of EVs in neurodegenerative disorders remain elusive, as there is still contradicting evidence about their function(s). EV nomenclature is not clearly established yet due to the lack of consensus on specific markers of EV subtypes^6^, hence here we will refer to small EVs (< 200 nm) as EVs. EVs have been reported to promote but also limit the aggregation of misfolded proteins in the brain^7–9^. These opposing results could be explained by the origin of EVs either from physiological tissues or malignant cell lines, which could bear different sets of proteins/enzymes.

Emerging evidence indicates that active proteases are associated with EVs^10,11^. Proteomic analysis has revealed the presence of matrix-metalloproteinases (MMPs) and sheddases, such as ADAMs, on the membrane of EVs^12^. Such EV-associated proteases have been shown to retain their enzymatic activity and can shed cell surface receptors^13^ and degrade substrates in the extracellular milieu, thus promoting extracellular matrix (ECM) remodeling. For example, the ECM degrading enzyme membrane type 1 matrix metalloproteinase (MT1-MMP) can be directed to EVs for extracellular release and can aid the degradation of type-1 collagen and gelatin^14^. With regards to neurodegeneration, clearance of extracellular amyloid beta (Aβ) has been shown to be mediated by the insulin-degrading enzyme (IDE) that is secreted in association with EVs^15,16^. In addition, EVs were shown to ameliorate the synaptic dysfunction caused by extracellular Aβ in vivo by sequestering toxic Αβ species on their surface^17^ and by accelerating the fibrilization of Αβ^18^. We focused on EVs as a potential modifier of extracellular α-syn and sought to assess their effect on α-syn degradation and fibrilization.

The association of α-syn-cleaving enzymes with EVs may represent a novel mechanism by which brain cells regulate α-syn levels in the extracellular space. Here we identified that among the key proteases implicated in this process are cathepsins, lysosomal enzymes involved in protein degradation and recycling. While prior studies suggest a connection between cathepsins and Parkinson’s disease (PD), the exact causal relationship remains unclear^19,20^. Experimental evidence indicates that cathepsins are upregulated in PD animal models, though earlier clinical studies found no significant differences in cathepsin activity between PD patients and controls^19^. Interestingly, cathepsins have also been detected in EVs^21,22^, and elevated levels of cathepsin D in neural-derived plasma EVs have been reported in Alzheimer’s disease^23^, suggesting a broader role for EV-associated proteases in neurodegeneration.

Degradation of extracellular substrates by EV-associated enzymes is an emerging area of research, and elucidating their role could provide critical insights into how they influence disease initiation and progression. Notably, EVs may store proteolytic activities within the extracellular matrix (ECM), releasing them in response to physiological stimuli during tissue remodeling—a potential new pathway for regulating pericellular proteolysis^14^.

Our findings support the hypothesis that EV-associated proteases may contribute to impaired extracellular α-syn clearance, raising the possibility of targeting these enzymes as a novel therapeutic approach for synucleinopathies.

## Results

### α-syn is readily degraded by brain EVs

**Fig. 1:**
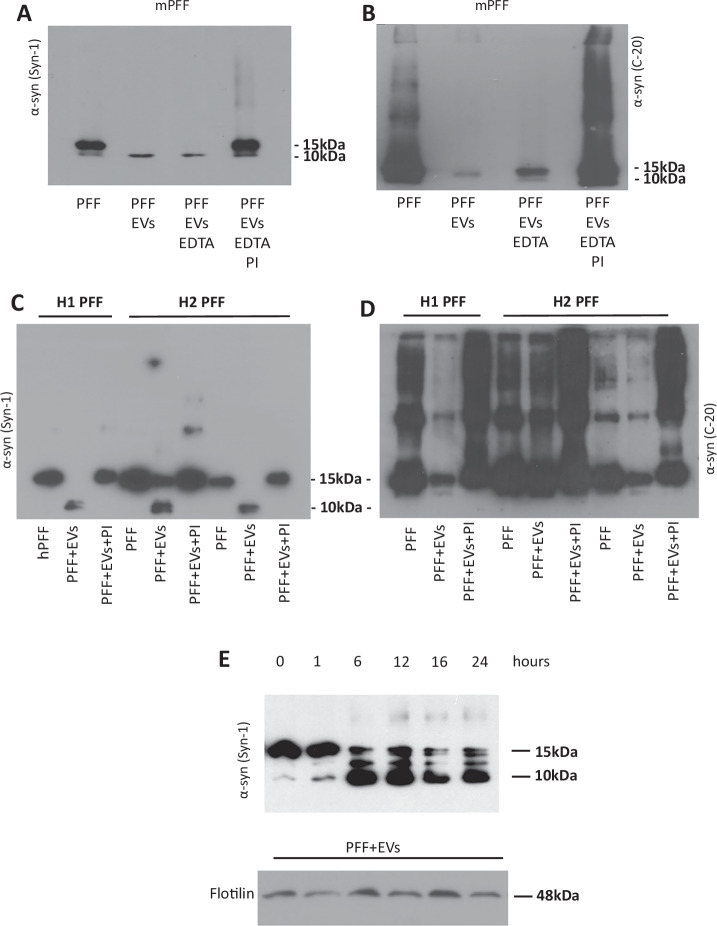
EVs cleave α-syn Preformed Fibrils (PFFs) in vitro. **A** Mouse PFFs (mPFFs) were incubated in vitro for 24 h at 37^o^C with and without EVs (Exo), and in the presence or absence of EDTA or EDTA with protease inhibitors cocktail (PIs) at a PFF:EV ratio 1:2. Immunoblotting with the Syn-1 antibody identified a truncated α-syn 10 kDa band upon incubation of mPFFs with EVs that was inhibited in the presence of protease inhibitors. **B** Western blotting of samples from (**A**) for α-syn with the C-20 antibody revealed that EVs degraded monomeric and oligomeric α-syn. The use of protease inhibitor cocktail inhibited the proteolytic effect of EVs on mPFFs and aided the formation of oligomeric α-syn species. **C** Human PFFs (two different preparations H1-H2 PFFs) were incubated as in (**A**) and (**B**) with EVs in the presence or absence of protease inhibitors and analyzed with Western blotting using the Syn1. As with the mPFFs, hPFFs were readily cleaved by the EVs in the same manner. **D** Western blotting of samples from (**C**) for α-syn with the C-20 antibody showed that inhibition of EV proteolytic activities resulted in increased formation of HMW α-syn species as with mPFFs. **E** Investigation of EV proteolysis in a time-dependent manner. hPFFs incubated with EVs displayed effective proteolysis at 6 h in a ratio of PFFs: EVs equal to 1:2. Staining for flotillin was used as a loading control for the EVs. Representative blots shown, *n* = 3.

Mouse α-syn PFFs (mPFFs) were exposed to brain EVs to investigate whether they could be cleaved by EV proteolytic activities. Brain EVs were isolated from α-syn KO mice to avoid interference from endogenously expressed α-syn. Immunoblotting with the Syn-1 antibody, which has high affinity for monomeric α-syn, revealed that EVs cleaved mPFFs and produced a prominent 10 kDa truncated α-syn product (Fig. 1A). The cleavage was completely inhibited in the presence of a protease inhibitor cocktail but not by EDTA, indicating that metalloproteases are not involved (Fig. 1A). The α-syn degradation capacity of the EVs was further confirmed with the C-20 antibody, which is specific for the *C*-terminus (aa 120–140) of α-syn. Incubation of mPFFs with EVs resulted in the reduction of the 15 kDa monomer with concomitant disappearance of high molecular weight (HMW) α-syn species (Fig. 1B). The truncated 10 kDa band that was detected with the Syn-1 antibody was absent with the C-20 antibody, suggesting that the cleavage is located at the *C*-terminus of the protein. Interestingly, inhibition of EV proteolysis by the cocktail of protease inhibitors resulted in the formation of HMW α-syn species. This indicates that in the absence of these proteolytic activities, EVs promote the aggregation of α-syn. As before, the cleavage was not inhibited by EDTA (Fig. 1B).

We then sought to investigate whether human α-syn species could also be degraded by purified mouse EVs. For this, we incubated EVs with human PFFs (hPFFs) from two different sources (described in “Materials and Methods”). Generated PFFs were characterized by EM and α-syn sedimentation assay as shown in Supplementary Fig. 1. As depicted in Fig. 1C, hPFFs were readily cleaved by EVs, and this cleavage was completely abolished in the presence of the protease inhibitor cocktail. As also seen with mPFFs, inhibition of α-syn cleavage aided the formation of HMW α-syn species (Fig. 1D). The EV proteolytic effect on α-syn hPFFs was examined in a time-dependent manner. As shown in Fig. 1E, a reduction of α-syn levels was evident at 6 h and appeared to reach a plateau thereafter.

**Fig. 2:**
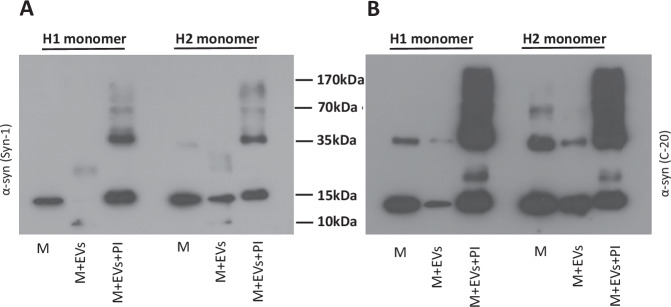
EVs cleave monomeric α-synuclein in vitro. Monomeric human α-syn (M) was incubated for 24 h at 37 °C with and without EVs, in the presence or absence of protease inhibitors (PIs). Two different preparations of α-syn were used (**A**) EV cleavage of monomeric α-syn, produces a 10 kDa truncated α-syn species as observed by western blotting with the Syn-1 antibody. Protease inhibitors cocktail (PI) stops the proteolytic cleavage but aids the formation of higher molecular weight species (HMW). **B** Western blotting with the C-20 antibody suggests C-terminus truncation of α-syn. Inhibition of proteolysis again induces the formation of HMW α-syn species. Both H1 and H2 α-syn PFFs behave in the same manner. Representative blots shown, *n* = 3.

We further investigated whether EVs could also degrade monomeric α-syn. To this end, we incubated human monomeric α-syn from two different sources (H1, H2) in the presence or absence of purified EVs. Similar to PFFs, α-syn monomer was readily cleaved by EVs, yielding a 10 kDa product that was detected by the Syn-1 antibody but not the C-20 antibody (Fig. 2A, B). The cleavage was again inhibited by the protease inhibitors cocktail. In accordance with the previous findings, the addition of protease inhibitors resulted in a striking formation of HMW α-syn species (Fig. 2B), suggesting that α-syn aggregation is promoted upon inhibition of EV proteolytic activities. EVs isolated from the conditioned medium of proliferating neuroblastoma SHSY5Y cells when incubated at the same ratio with hPFFs for 24 h at 37 °C also retained this ability to degrade hPFFs (Supplementary Fig. 2).

### Mapping of α-syn cleavage sites

**Fig. 3:**
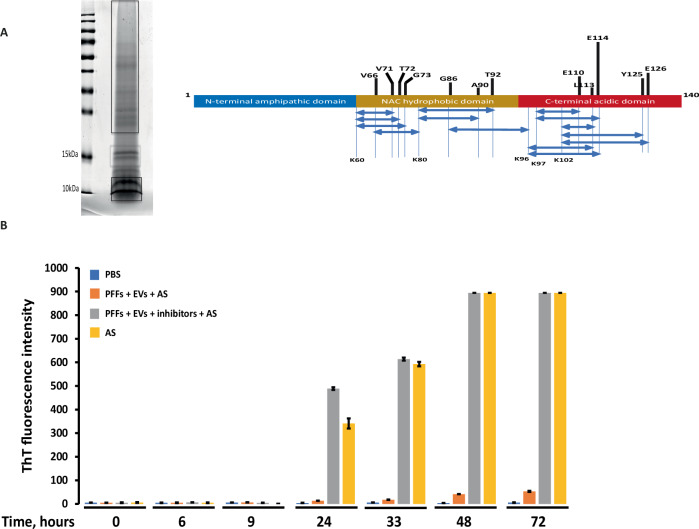
EV-generated α-syn fragments have reduced seeding capacity. **A** Coomasie blue stain of the EV-cleaved α-syn. Brackets indicate MW bands that were gel excised for proteomic analysis. The identified cleavage sites are depicted above the schematic representation of the α-syn molecule. The length of the bar represents the frequency of this cleavage based on the MS/MS spectra. The higher the length, the more common. Thus, the E114 is the most common cleavage site. Other cleavage sites are found inside the NAC domain or at the C-terminus. Below the schematic structure of the α-syn molecule the Lys are marked, and the double-headed arrows represent the peptides that were identified in MS/MS. Cleavage at Lys is due to the semitryptic digest, while cleavage at other positions is due to proteases found in EVs. Cleavages found in only one MS/MS have not been included. **B** Seeding of monomeric α-syn is inhibited in the presence of hPFFs pre-treated with EVs. Monomeric human α-syn was incubated with hPFFs pre-treated with EVs (orange) and hPFFs pre-treated with EVs in the presence of protease inhibitors (gray). Aggregation kinetics of reactions were monitored over time through ThT fluorescence measurements. PBS (blue) or monomeric α-syn as seeds (yellow) were used as negative and positive controls, respectively. hPFFs pre-treated by EVs suppressed the aggregation of monomer α-syn, while in the presence of protease cocktails inhibitors, the aggregation was promoted. Data are expressed as mean ± standard deviation (SD) of 3 independent experiments. *P ≤ 0.05; **P ≤ 0.01; ***P ≤ 0.001; ****P ≤ 0.001.

Mouse α-syn PFFs were incubated with EVs as described, and the products were resolved on SDS-PAGE. The bands corresponding to ~10 kDa and to HMW species were cut, subjected to tryptic digestion, and analyzed with LC-MS/MS. Peptide fragments that are produced through cleavage at sites other than lysine, correspond to cleavages by the proteolytic activities present on EVs and the identified cleavage sites are depicted in Fig. 3A. Based on peptide abundances, the site E114 appears to be the most common of the highest preference (Fig. 3A). Cleavage at E114 results in C-terminal truncation, which is consistent with the antibody mapping.

### Processing of hPFFs by EVs produces fragments that lack seeding capacity

The seeding capacity of hPFFs pre-incubated with EVs was tested by ThT assay following incubation with monomeric α-syn. The intensity of ThT fluorescence increased only slightly after 2 and 3 days (Fig. 3B, orange bars), which indicates that EV-derived PFF proteolytic fragments likely inhibit the aggregation of α-syn monomers. In contrast, when the protease inhibitor cocktail was added, a fast aggregation kinetics for monomeric α-syn was observed (Fig. 3B, gray bars). Taken together, these findings suggest that (a) the proteolytic fragments produced from the processing of hPFFs by EVs do not act as seeds for the templating and aggregation of monomeric α-syn and (b) the EVs, with quenched enzymatic activities, promote the aggregation of α-syn (Fig. 3B).

### Profiling of EV proteolytic activities responsible for α-syn cleavage

**Fig. 4:**
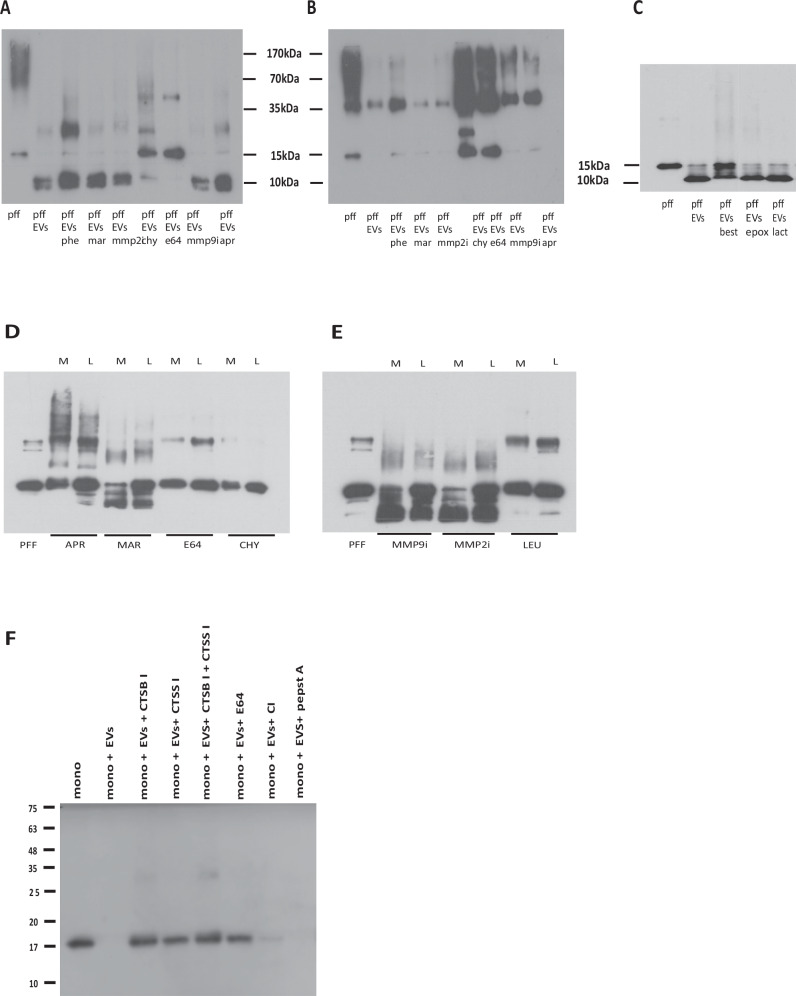
Inhibitor-based profiling of EV-associated proteases responsible for α-syn cleavage. **A**–**C** Human α-syn PFFs (hPFFs) were incubated with EVs at 1:2 ratio at 37 °C for 24 h and analyzed by Western blotting using the Syn-1 in A and C or C-20 antibodies in (**B**). **D**, **E** Mouse α-syn PFFs (mPFFs) were incubated with either membranous (M) or luminal (L) EV fractions and analyzed by Western blotting using the Syn-1 antibody. Proteolytic activities of different specificities were abolished by addition of corresponding specific inhibitors as following: 1,10 phenanthroline (phe), the broad-spectrum matrix metalloprotease inhibitor marimastat (mar), inhibitor specific for MMP2 (mmp2i), chymostatin (chy), a cysteine protease inhibitor E-64, inhibitor specific for MMP9 (mmp9i), aprotinin (apr), leupeptin (leu), epoxomicin (epox), lactacystin (lact), bestatin (best) and pepstatin A (pepstatA). **F** 100 ng monomeric α-syn (mono) (lane 1) were incubated at 37 °C for 21 h with 200 ng EVs (exo) (lane 2) in the presence and absence of specific protease inhibitors. The 8-hydroxy-5-nitroquinoline specific inhibitor of cathepsin B (CTSB I, lane 3) and LHVS inhibitor of cathepsin S (CTSS I, lane 4) were also added in combination (lane 5). The E-64 general inhibitor of cysteine proteases (lane 6), the calpain inhibitor I (CI, lane 7), and cathepsin D inhibitor pepstatin A (lane 8) were also tested at the concentrations mentioned above. Two different preparations of EVs were used in quadruple experiments. C20 was used for the detection of α-syn.

Τo gain insight into the protease(s) responsible for α-syn cleavage, we examined the effects of different protease inhibitors (cysteine, serine, metalloprotease) on cleavage of hPFFs by EVs by immunoblotting using the Syn-1(Fig. 4A) and C-20 (Fig. 4B) antibodies. The proteolytic cleavage of α-syn was abolished by addition of E-64, a universal cysteine protease inhibitor, whereas it was not affected by the broad-spectrum MMP inhibitor marimastat, 1,10-phenanthroline, and the MMP2 and MMP9 specific inhibitors. The overall proteolytic activity remained unaffected by aprotinin (Fig. 4) and AEBSF (not shown), indicating that serine proteases are not involved in the cleavage of α-syn. The proteasomal inhibitors epoxomicin and lactacystin, as well as the reversible aminopeptidase inhibitor bestatin, did not affect the cleavage (Fig. 4C). Taken together, these data suggest that the protease(s) associated with EVs, which are responsible for cleavage of α-syn hPFFs, belong to the cysteine protease family.

Further, EVs were sonicated and then fractionated to luminal and membranous fractions, as described in Materials and Methods. Incubation of mPFFs (100 ng) with either the membranous (200 ng) or luminal (200 ng) EV fractions for 24 h at 37 °C resulted in the efficient degradation of the PFFs. We also performed inhibitory profile experiments to determine which protease activities were present in the 2 EV fractions. As depicted in Fig. 4D, E, the proteolytic activity was abolished by the addition of E-64, chymostatin, and leupeptin in both fractions, whereas it was not affected by marimastat and specific MMP2 and MMP9 inhibitors.

### Proteomic characterization of EVs and identification of candidate proteases

To investigate further the protein content of EVs and, potentially, identify proteases that could account for the observed cleavage of PFFs, we used a bottom-up proteomic analysis. EVs were isolated from adult α-syn KO mice, as described. Three biological replicates were performed in technical triplicate. In total, the identified dataset comprised 3237 proteins. After filtration of proteins for potential contaminants, reversed hits, and proteins that were only identified by site, we concluded in a working list of 2221 proteins with a unique accession number, which was used for further analyses.

First, we examined the enrichment of our proteomic dataset in EVs to verify their purity. For this purpose, we compared our dataset (Supplementary Fig. 3A) to the Vesiclepedia database and found 73 of the top 100 Vesiclepedia’s markers to be present in our dataset. Further comparison with the Exocarta, an EV-specific database, revealed an even higher overlap with our dataset (77%), suggesting that the isolated EVs are enriched in EV (Supplementary Fig. 3A). In the same context, following gene ontology analysis using the DAVID platform EVs (termed exosomes) were identified as the top hit cellular component (Supplementary Fig. 3D).

EM and nanoparticle-tracking analysis (NTA) verified the presence of disc-shaped nanovesicles with a size ranging between 50 and 250 nm characteristic of EV (Supplementary Fig. 3B, C). These results confirmed that our proteomic datasets were indeed enriched in EV. The integrity of the EV was also verified using Western immunoblotting for known EV markers (Supplementary Fig. 3E).

Candidate proteases were searched in the identified proteomic dataset with the MEROPS *Mus musculus* peptidase database (release 12.4). As shown in Supplementary Table II, 89 proteases were identified. Interestingly, this set contained all the proteasomal components, suggesting that active proteasome is a cargo in our EVs. Nevertheless, this list was manually annotated to remove potential proteins that bear protease domains but are not true proteases and identify the classes of protease specificities that are compatible with the determined inhibition profile. Given that serine protease inhibitors, metalloprotease inhibitors, and proteasome inhibitors had no effect on α-syn and PFFs cleavage by EVs it can be concluded that serine proteases, metalloproteases, and the proteasome are likely not involved. Thus, these proteases were excluded from the list of 89 proteases and the candidate-proteases narrowed down to the five (5) proteases that could account for PFF cleavage shown in Table 1. Interestingly, these included cathepsins that have been implicated in the cleavage of α-syn^24,25^.

**Table 1:** List of aspartyl and cysteine proteases present in EVs

Gene Symbol	Protein	Activity specificity
*Ctsd*	Cathepsin D	aspartyl
*Ctss*	Cathepsin S	cysteine
*Ctsb*	Cathepsin B	cysteine
*Capn2*	Calpain 2	cysteine
*Capn5*	Calpain 5	cysteine

### Validation of the protease(s) involved

Enzymatic assays showed that brain EVs carry active proteases based on the efficient cleavage of the Z-Phe-Arg-AMC substrate (Supplementary Fig. 4, blue), which is used to determine the activity of lysosomal cathepsin enzymes but also cleaved by other proteases (plasmin, kalikreins, etc), therefore suitable to monitor the overall proteolytic activities. The activity of each enzyme found in EVs (Table 1) was dissected by use of a specific inhibitor. It turns out that active cathepsin D is present in EVs, since substrate cleavage was inhibited about 20% by the cathepsin D inhibitor pepstatin A (brown). Nevertheless, the proteolytic activities of EVs were completely abolished by the E-64 inhibitor of cysteine proteases (cyan), like the cathepsins B and S. Proteolysis rates were strongly reduced by the cathepsin B (red) and cathepsin S (pink) inhibitors, providing evidence that these enzymes are active in EVs.

To identify which of the candidate protease(s) are indeed involved, monomeric α-syn was cleaved by EVs, and specific inhibitors of the identified proteases were added either alone or in combinations. EV-cleaved α-syn, as shown in Fig. 4F (lane 2); however, this cleavage was highly inhibited in the presence of a cathepsin B specific inhibitor. The cathepsin S inhibitor LHVS abolished cleavage significantly although to a lesser extent. It should be noted that LHVS inhibits cathepsin S selectively at nM concentrations (Ki = 5.9 nM); it can also inhibit cathepsin B but at μM concentrations (Ki = 39 μΜ). Degradation was completely inhibited by the combination of cathepsin B and S inhibitors Fig. 4F (lane 5), indicating that these two proteases are solely responsible. The fact that cleavage of α-syn was not affected neither by the calpain inhibitor nor by pepstatin A excludes calpains and cathepsin D as the α-syn processing proteases in this context.

### EVs-generated proteolytic fragments of α-syn do not augment seeding of endogenous α-syn in primary cortical neurons

**Fig. 5:**
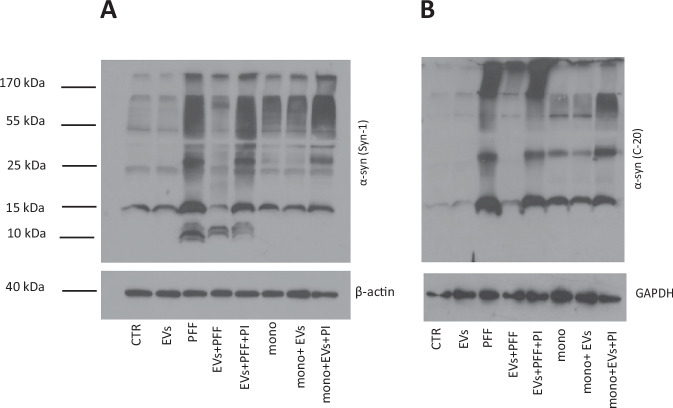
EV-induced proteolysis of human α-syn PFFs produces species that do not aid the aggregation of endogenous α-syn. Primary cortical neurons (5div) were treated for 72 h with hPFFs, hPFFs pre-incubated with EVs (Exo) in the presence or absence of protease inhibitors (PIs), as well as EVs alone. Similar incubations were used with monomeric α-syn (mono). Untreated cells were used as control (CTR). **A** Immunoblotting for α-syn with the Syn-1 antibody revealed that EV association of PFFs produces fragments that do not augment α-syn aggregation. Beta-actin was used as loading control. **B** Immunoblotting for α-syn with the C-20 confirmed the inability of the EV-degraded PFFs to seed the aggregation of α-syn in neuronal cultures. GAPDH was used as loading control.

We next sought to examine the impact of the observed EV proteolysis on the pathogenicity of the cleaved hPFFs, specifically, on the seeding of endogenous α-syn. Initially, we examined the effect of EV proteolysis of hPFFs on primary mouse cortical neurons. Specifically, cortical neurons were treated with a) EVs alone, b) hPFFs alone, c) hPFFs pre-incubated with EVs, d) hPFFs pre-incubated with EVs in the presence of protease inhibitors. Identical incubations were performed using monomeric α-syn. 3 × 10^5^ neuronal cells were treated with 1 μg of monomeric α-syn or hPFFs for 72 h (see “Materials and Methods”). Cells were then collected, lysed, and analyzed for α-syn by immunoblotting. As expected, treatment of neurons with hPFFs led to the formation of intracellular HMW α-syn species (Fig. 5A). When hPFFs were pre-incubated with EVs oligomeric species were significantly reduced (Fig. 5A). However, addition of protease inhibitors reinstated the appearance of intracellular HMW α-syn species. The above results were verified using the C-20 antibody (Fig. 5B). To further prove that the observed effect was on the endogenous α-syn we used the phospho α-syn specific antibody to examine endogenous levels of the phosphorylated protein in our cortical cultures, considering that recombinant PFFs cannot be phosphorylated in vitro. As expected, treatment with PFFs increased the levels of endogenous α-syn phosphorylation, an effect that was abolished in the presence of EV-cleaved PFFs (Supplementary Fig. 5). Similar results were obtained when neuronal cells were treated with monomeric α-syn, indicating that monomeric α-syn formed oligomeric species when EV proteolysis was inhibited (Fig. 5A).

**Fig. 6:**
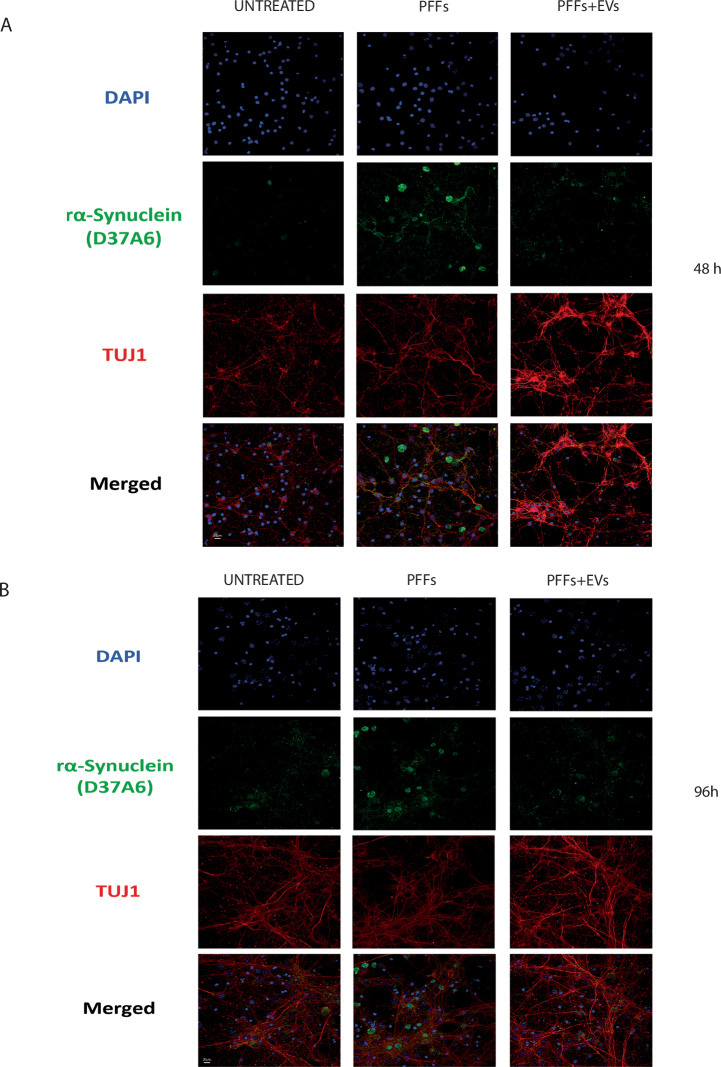
Truncated α-syn species originating from PFFs proteolysis by EVs do not trigger the recruitment and aggregation of the endogenous α-syn in recipient neurons. Confocal microscopy images of primary cortical neurons labelled for endogenous mouse α-syn (rα-Synuclein D37A6), 5 days in vitro (DIV), neuronal cells were treated with PFFs or EVs-cleaved PFFs for (**A**) 48 h and (**B**) 96 h. Untreated cells were used as control. TUJ1 and DAPI were used to stain neurons and neuronal nuclei, respectively. Scale bar represents 50 μm.

We further explored the seeding effect of EV-associated α-syn species on the recipient cells *via* immunofluorescence. Staining for the endogenous mouse α-syn with the rodent specific (rα-Synuclein D37A6) antibody revealed the expected aggregation of the endogenous α-syn 48 h following treatment with hPFFs. Such an effect was not observed when cells were incubated with hPFFs exposed to EVs, suggesting that the produced truncated α-syn species do not have the ability to act as seeds to recruit the endogenous α-syn for aggregation (Fig. 6A). Similar results were obtained 96 h post-treatment (Fig. 6B).

**Fig. 7:**
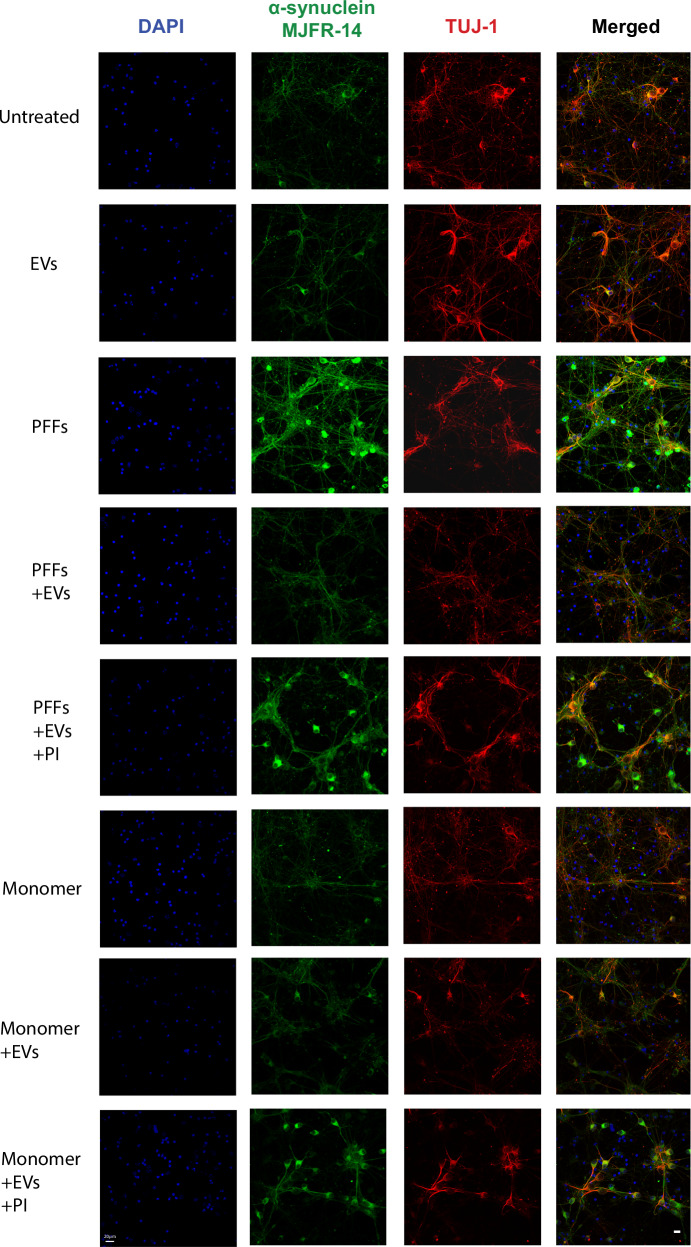
EV-associated proteolysis of α-syn does not induce the formation of aggregated α-syn species in neurons. Confocal microscopy images of primary cortical neurons labelled for aggregated α-syn (MJFR-14). Cells were treated at 5 DIV with hPFFs, hPFFs pre-incubated with EVs in the presence or absence of protease inhibitors, as well as with α-syn monomers pre-incubated with EVs in the presence or absence of protease inhibitors (PIs), for 96 h. Untreated cells, as well as cells treated only with EVs were used as controls. TUJ1 and DAPI were used to stain neurons and neuronal nuclei, respectively. Scale bar represents 20 μm.

To further verify that EV-cleaved PFFs are incompetent in seeding endogenous α-syn, we assessed α-syn aggregation using the MJFR14 oligomer-specific antibody. Neuronal cells were treated with hPFFs or EV-cleaved hPFFs for 96 h. As shown in Fig. 7, immunostaining with the MJFR14 revealed increased levels of aggregated α-syn were formed when cells were treated with hPFFs but not with EV-cleaved PFFs. However, hPFFs pre-incubated with EVs in the presence of the protease inhibitor cocktail retained their ability to seed α-syn (Fig. 7). Similar results were obtained when the neuronal cultures were treated with monomeric α-syn. Again, EV-cleaved α-syn monomer did not affect oligomerization of endogenous α-syn. Nonetheless, when EV cleavage was arrested by the protease inhibitor cocktail, monomeric α-syn aided the formation of oligomeric species (Fig. 7). These data indicate that EVs in the absence of proteolytic activities promote the aggregation of α-syn monomer that facilitates its uptake.

### EVs-generated proteolytic fragments of α-syn exhibit reduced pathology following intrastriatal injections in vivo

**Fig. 8:**
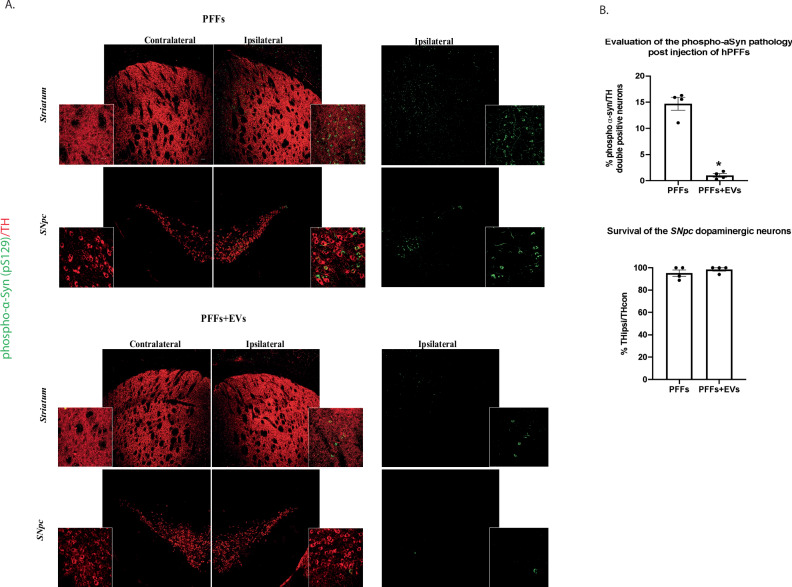
Injection of EV-cleaved PFFs causes significantly reduced α-Syn pathology in WT mice, compared to non-cleaved PFFs, at 1 month post inoculation. **A** Representative confocal images of the contralateral and ipsilateral striatum and SNpc of uncleaved PFF- (left panel) and EV-Cleaved PFF-injected (right panel) mice, 1-month post-injection, showing double immunostaining of phosphorylated-α-Syn (green) and tyrosine hydroxylase (TH, red). [(low magnification; 10x, scale bar 50 μm and high magnification; 63x, scale bar 20 μm)]. **B** Graph illustrating the estimated α-Syn pathology in the ipsilateral SNpc, at 1-month post-intrastriatal injections, expressed as % of double positive phospho-α-Syn/TH neurons, normalized to the total number of dopaminergic neurons (upper panel). Graph depicting the stereological counts of the nigral dopaminergic cell somata, estimated as the percentage of TH positive neurons of the ipsilateral side versus the ones found in the contralateral side (lower panel). (*n* = 4 animals per group). Data represent mean values ± SEM. Differences were estimated using nonparametric Mann-Whitney.

To examine the toxic potential of the EV-cleaved hPFFs in vivo, we stereotactically injected the right dorsal striatum of adult wild type mice with 2.5 μg of PFFs incubated overnight at 37 °C in the presence or absence of EVs. The presence of phosphorylated α-syn inclusions was determined by immunofluorescence at 1 month post injection (Fig. 8A). Similar to our in vitro findings, EV-cleaved hPFFs were significantly less potent in inducing α-syn pathology in comparison to uncleaved hPFFs, as exhibited by the reduced numbers of TH neurons bearing phospho α-syn inclusions (1.02% ± 0.34 vs 14.7% ± 1.22, Fig. 8B). As expected, in the ipsilateral striatum the respective phospho α-syn pathology was reduced, following inoculation with EV-cleaved PFFs (Fig. 8A). The observed phospho-α-syn inclusions, in both groups, were also positive for the conformation-specific antibody SynO2, with affinity for the aberrant forms of the protein^26^ (Supplementary Fig. 6). Importantly, following 1-hour treatment at 37 °C with PK (1 h), nigral α-Syn accumulations were still immuno-reactive for the rodent-specific antibody D37A6 (Supplementary Fig. 6), verifying the presence of insoluble aggregates.

Finally, no profound nigral neuronal loss was observed following PFF-inoculation at one month-post injection, in accordance to literature^27–30^. As expected, following stereological counting, we did not observe any significant differences in the survival of the *SNpc* dopaminergic neurons between mice injected with PFF or EV-cleaved PFFs (Fig. 8B).

### Cathepsin S is a processing protease of human α-synuclein

Proteolytic profiling of mouse brain EVs revealed that active cathepsin S is present and cooperates with cathepsin B in proteolytic processing of α-syn. Thus, we investigated whether the human cathepsin S can also recognize as a substrate and cleave the monomeric α-syn. Indeed, as shown in Supplementary Fig. 7 (lane 2), we observed complete cleavage of α-syn by enzymatically active cathepsin S, which was fully inhibited upon addition of the LHVS inhibitor of cathepsin S activity (Supplementary Fig. 7, lane 3).

## Discussion

Given that the α-syn is physiologically secreted, it is feasible that disruption of its canonical degradation and clearance from the extracellular milieu could contribute to the PD process. We and others have shown that α-syn is associated with EVs, and this association can amplify pathology transmission in the brain^5,31–33^. However, the exact role of this interaction between EVs and α-syn is not elucidated, and whether EVs could play an important role in initiation and progression of PD pathology is still unclear. Here, we show that purified intact brain EVs can modify the levels of α-syn via a yet undescribed proteolytic mechanism. When neurotoxic recombinant α-syn PFFs^34^ were exposed to brain EVs, PFFs were cleaved and α-syn species with molecular weights below 15 kDa were produced proteolytically, since a universal protease inhibitor cocktail prevented the cleavage of PFFs completely and, interestingly, triggered the formation of additional HMW α-syn species. Inhibitor profiling of EVs proteolytic activities showed that the E-64 general inhibitor of cysteine proteases completely abolished cleavage of α-syn PFFs by EVs and the formation of HMW species pointing to cysteine protease(s) as major candidate(s). Serine protease or metalloprotease inhibitors, but also proteasome inhibitors, had no effect. Similarly, monomeric α-syn was readily cleaved by brain EVs, and protease inhibitors blocked this cleavage and induced the formation of HMW α-syn species. Antibody detection showed that the EV-induced truncation mainly occurred at the *C-*terminus. Of note, the EV-cleaved PFFs inhibited the aggregation kinetics of monomeric α-syn monitored by ThT fluorescence, indicating that EVs could affect the aggregation of monomeric α-syn, which is a critical step in the formation of toxic species.

α-syn truncation and aggregation are tightly linked with its neurotoxicity. In this respect, when primary cortical neurons were treated with PFFs, HMW species of the endogenous α-syn were formed; however, this was not observed with EV-treated PFFs. Strikingly, in the presence of protease inhibitors EV-treated PFFs were able to seed the endogenous α-syn, leading to formation of HMW α-syn species. The effect of EV proteolysis was quite similar with monomeric α-syn. The aggregation of the endogenous α-syn was monitored by rα-syn D37A6 rodent specific antibody staining but also with the α-syn oligomer-specific MJFR antibody, which further confirmed that the truncated α-syn species resulting from EV-induced proteolysis do not have the ability to act as seeds and recruit the endogenous α-syn for aggregation. This was also verified in vivo, where EV-cleaved PFFs showed significantly reduced ability to induce endogenous α-syn pathology at 1 month post injection. It is possible that altered EV-associated proteolytic activity may affect α-syn levels and hence aid its accumulation and seeding ability. Collectively, these results suggest that perturbed proteolytic activities of brain EVs may be critical for disease initiation and progression. To this end, another report by Tan and colleagues^35^ suggested that newly synthesized cathepsins are sorted to EVs, suggesting that alterations in this mechanism may contribute to altered EV biogenesis, secretion, and function.

The proteome of our purified EVs was analyzed by mass spectrometry to identify all their proteases, which included enzymes of all protease classes (Supplementary Table I). A predominant feature of the EV proteome was the presence of all the components of the 20S proteasome, which were identified with very high confidence level. In this respect, it was demonstrated that a functional proteasome co-purified with mesenchymal stem cells EVs correlated with a modest but significant reduction in oligomerized Aβ protein in a mouse model of myocardial infarction^36^. The presence of all seven α-subunits and all seven β-subunits of the 20S core particle in our brain EVs indicates that they contain 20S proteasome enzymatic activity. However, the specific and irreversible 20S proteasome inhibitor epoxomicin, as well as lactacystin, failed to inhibit the observed α-syn cleavage by EVs, suggesting that the proteasomal subunits present on EVs are not involved.

By enzymatic assays and inhibitor profiling, the candidate proteases were narrowed down on cathepsins B and S, as the EV proteases involved in α-syn cleavage. Notably, both cathepsin S and B have been shown to be active in a neutral extracellular pH^37,38^. Cathepsin B (among other enzymes) was shown to be inhibited by chymostatin but also by the 8-hydroxy-5-nitroquinoline specific inhibitor for cathepsin B. Profiling of cleavage sites by mass spectrometry identified E114 as a major site of α-syn proteolysis, which is reported to be the site recognized by cathepsin B^39^. Lysosomal proteases including cathepsin D^25,40^, as well as cathepsins B and L^24^ were previously associated with the degradation of α-syn. In a similar context, cathepsin B has been shown to be associated with regulatory secretory vesicles of neuronal cells, where it was shown to increase the production of amyloid beta (Aβ) by cleavage of the amyloid precursor protein (APP) at the β-secretase site^41^. Using purified mouse brain and liver lysosomal extracts and human cathepsins, McGlinchey and Lee demonstrated direct interaction between α-syn and cathepsins B and L^24^, analyzed via LC-MS peptide mapping. In a recent study, we showed that brain derived EVs isolated from the PFF mouse model of PD exhibit significantly reduced levels of cathepsin B and are able to transmit α-syn pathology^27^.

Notably, here, we identified cathepsin S as a novel α-syn processing enzyme. Cathepsin S has been shown to be present in EV derived from microglial cell lines^42^. We show that cathepsin S is present in brain EVs, and cleavage of α-syn and PFFs by EVs is significantly inhibited by the LHVS inhibitor at nM concentrations, at which it is a selective inhibitor for cathepsin S.

Cumulatively, we demonstrate that brain EVs contain functional cellular proteases that can be secreted from cells in association with the endosomal pathway and provide strong support for a “protective” role of EVs in α-syn pathology transmission. We further show that the EV-induced cleavage of α-syn is driven jointly by cathepsin B and cathepsin S proteases. Moreover, the resulting truncated species lack the ability to act as seeds for recruitment and further aggregation of the endogenous α-syn. In recent years, proteolytic processing of extracellular α-syn has emerged as a new significant field for active investigation with putative implications for disease therapies. Our results argue that EV-associated cathepsins B and S may play a pivotal role towards this direction. Dysfunction of EV proteases in disease could lead to ineffective clearance of α-syn. To this end, neurodegenerative diseases such as Alzheimer’s, Parkinson’s, or prion disease that are caused by accumulation of denatured or misfolded proteins could be alleviated by EVs carrying functional proteases.

## Methods

### Isolation and characterization of brain EVs

Whole brain EVs were isolated from adult α-syn knockout (KO) mice (C57BL6/JOlaHsd mice, Harlan Laboratories) as previously described^34,43^, with some modifications. We chose to isolate EVs from α-syn KO mice, to exclude interference of endogenous α-syn present in EV cargo. Briefly, excised brains hemispheres were homogenized and enzymatically dissociated with papain (20 units/ml, Worthington) at 37 °C for 30 min (7 ml/brain). Papain was previously diluted in fetal bovine serum (FBS)-free Dulbecco’s modified Eagle’s media (DMEM), containing cysteine (1 mg/ml) and 1% (v/v) penicillin/streptomycin (P/S). Enzymatic digestion was terminated by addition of two-volumes of ice-cold FBS-free DMEM, containing 1% P/S. Tissue lysate was passed through the tip of a 10 ml plastic pipette until the solution was homogenous, then centrifuged at 300 × *g* for 10 min at 4 °C. Supernatant was initially passed through a 40 μm mesh filter and then through 0.45 μm and 0.2 μm filters. Filtrate was sequentially centrifuged at 2000 × *g* for 10 min at 4 °C, 10,000 x *g* for 30 min at 4 °C and finally at 100,000 × *g* for 70 min at 4 °C. Following aspiration of the supernatant, pellet from final centrifugation was diluted in 1.5 ml of 0.95 M sucrose solution and then loaded onto a sucrose step gradient column containing six steps: 0.2, 0.6, and 0.95 M (containing the EV enriched resuspended pellet), 1.3, 1.6, and 2 M. Sucrose gradient column was centrifuged at 200,000 × *g* at 4 °C for 16 h. Seven fractions were separated according to the gradient (a–g from top to bottom). Fractions B, C, and D were collected individually, diluted in PBS up to 22 ml of final volume, and centrifuged at 100,000 x *g* (70 min, 4 °C). The resulting pellets were re-suspended in PBS and stored at −80 °C until used. Fraction d was used for subsequent assays. EV enrichment in the EVs was assessed through measurement of the acetylcholinesterase activity as described^44^ immunoblotting against EV- specific markers, and electron microscopy. The total protein load of the EV fractions was measured by the Bradford assay.

### Electron microscopy (EM)

Vesicle, as well as PFF EM was carried out as described in Melachroinou et al.^27^. Briefly, vesicles were fixed with 4% paraformaldehyde (PFA) in 100 mm Na_2_HPO_4_, applied to a 300-mesh copper grids with carbon-coated formvar film, and stained with uranyl-oxalate and methylcellulose-uranyl acetate. Human PFFs (5 μl) were loaded on Formvar-coated 400 mesh copper grids, fixed with 0.5% glutaraldehyde, and stained with 2% uranyl acetate (Sigma-Aldrich, USA). Images were obtained using a Philips 420 Transmission Electron Microscope (acceleration voltage of 60 kV) and captured with a Megaview G2CCD camera (Olympus SIS, Münster, Germany) and iTEM image capture software.

### Nanoparticle tracking analysis (NTA)

NTA of EV preparations was performed as previously described^27^ using NanoSight NS300 instrument (Malvern Instruments, Amesbury, UK) equipped with a 532 nm laser (green), a high sensitivity sCMOS camera. The analysis of the acquired videos was with NanoSight NTA 3.4 build 3.4.4 software. A total of 1500 frames were examined.

### EV fractionation

EV proteins were separated to membranous and luminal fractions, as described before^45,46^ with Triton-X treatment, with minor modifications. Briefly, EV were subjected to three 15 min dry-ice freezing-thawing cycles, to mechanically lyse the EV and release their luminal content. Subsequently, EV suspension was treated with 2% w/v Triton-X, containing 10 mM Tris-HCl 7.5, and 150 mM NaCl. The suspension was incubated on ice for 30 min with frequent vortexing. Subsequently, EV were centrifuged at 50,000 × *g* at 4 °C, overnight to separate the insoluble and soluble fractions.

### Animals and ethical approval

Mice were housed in individually ventilated cages with free access to food and water, under a controlled light-dark cycle (12 h light–12 h dark) and temperature (21 + 1 °C), at the animal facility of the Biomedical Research Foundation of the Academy of Athens. All experimental protocols were performed in accordance with the EU directive guidelines for the care and use of experimental animals (86/609/EEC; 27/01/1992, No. 116) and were approved by the Institutional Ethics Committee for Use of Laboratory Animals, and an authorized veterinarian committee in accordance to Greek legislation (Presidential Decree 56/2013, in compliance with the European Directive 2010/63).

### Primary cortical neuron cultures

Primary cortical neurons were prepared from wild-type (wt) mouse embryonic brains isolated on E16 as described in Karampetsou et al.^28^. Cells were plated in 24-well cell culture plates coated with poly-D-Lysine (PDL) (0.1 mg/mL) and cultured in BrainPhys neuronal medium, containing 2% SM1 neuronal supplement (STEMCELL Technologies), 0.5 mM of L-glutamine and 1% (v/v) P/S. Approximately 9 × 10^4^ cells/12 mm coverslip were plated for immunofluorescence experiments and 3 × 10^5^ cells/6-well for immunoblotting. Cells were kept in a humidified incubator with optimal cell culture conditions of 37 °C and 5% CO_2_ and were supplemented with 100 μl/well of the neuronal medium every two days.

Neuronal cell cultures were treated with products from in vitro proteolytic reactions, i.e., PFFs alone or PFFs with EVs in the presence or absence of protease inhibitors, incubated for 24 h at 37 °C, as well as α-syn monomer controls. Treatments of the neuronal cell culture took place during day 5, depending on cell fitness. Neurons were treated with 1 μg of α-syn per 3 × 10^5^ cells for 48, 72, and 96 h.

### Stereotaxic injections

Unilateral striatal injections were performed in 3–4 months old WT C57BL6/C3H mice, as previously described^27^. The coordinates used for the targeting of the right dorsal striatum from bregma were: anteroposterior of +0.5 mm, mediolateral of +2.0 mm, and dorsoventral in two depths of 3.2 and 3.4 mm (“The Mouse Brain in Stereotaxic Coordinates”, G.Paxinos & K.B.J. Franklin). 2.5 μg of hPFFS or equal amount of exosome-cleaved hPFFs were diluted to a final volume of 3 μl of phosphate-buffered saline (PBS) (4 mice/ group). Mice were sacrificed at one-month post-injection, for the estimation of phosphorylated α-syn (phospho-α-syn) pathology by immunohistochemical analysis.

### Stereology

For the assessment of the survival of the *SNpc* dopaminergic neurons, sterological counting was performed, as previously described by Melachroinou et al.^27^. Briefly, ten 30 μm thick sections with a 3-section interval, along the rostrocaudal axis (four mice per group) were stained for TH. Following incubation with the secondary antibody (Vectastain Elite ABC kit, Vector labs), 3,30- diaminobenzidine (DAB; Dako) was used as chromogen. The total number of the TH dopaminergic neurons in the *SNpc* was counted using the Stereo Investigator v10.0 software (MBF Bioscience, United States). A 2.5× and 63× glycerol immersion objectives were used to generate the counting contours and count the neurons, respectively. (Optical dissector height:12 μm, grid size: 100 μm, counting frame: 50 μm, acceptable coefficient of error: Gundersen, *m* = 1 of ≤ 0.1).

### Immunofluoresence

Primary cortical neurons grown in PDL-coated glass coverslips were fixed with 4% sucrose in PBS and 4% PFA for 15 min at room temperature (RT). For immunocytochemistry, fixed cells were incubated first in blocking buffer, PBS containing 3% bovine serum albumin (BSA) and 0.1% Triton, for 20 min at room temperature, then with the primary antibodies diluted in blocking buffer for 16 h at 4 °C. Following four washes in PBS, secondary antibodies diluted in blocking buffer were added for 1 h at RT, and coverslips were kept in the dark. Cell nuclei were stained with DAPI (0.5 μg/ml). After final PBS washes, the coverslips were mounted on a slide using Vectashield (Vector-labs). Images were obtained with Leica SP5 upright confocal laser scanning microscope and presented with ImageJ/Fiji v 2.0.0 software.

### Immunohistochemistry and image analysis

Mice were intracardially perfused, under isoflurane anesthesia, with ice-cold PBS, followed by 4% paraformaldehyde in PBS, as previously described^27^. Following overnight post-fixation at 4 °C, brains were dehydrated by sequential incubation in 15% and 30% sucrose in PBS at 4 °C, snap-frozen into iso-pentane (−45 °C), and 30 μm free-floating cryo-sections were collected. Coronal sections covering the whole ventral midbrain were washed trice (3x10min) with PBS, blocked for 1 h in 5% normal goat serum (NGS) in PBS containing 0.1% Triton X (blocking buffer), and incubated with the primary antibodies anti-tyrosine hydroxylase (TH) (mouse monoclonal, 1:2000, Merck- Millipore) and anti-phospho-S129 α-Syn (rabbit monoclonal, 1:2000, Abcam), in blocking buffer for 24 h at 4 °C. Following washing with PBS, sections were incubated for 2 h at RT, in blocking buffer containing the respective secondary antibodies (Invitrogen). Finally, sections were mounted on Poly-D-lysine slides (VWR) using Dako fluorescent mounting medium (Dako). Images were obtained with Leica SP5-II confocal microscope, and Fiji v2.0.0 software was used to perform background subtraction and thresholding. For the estimation of phospho-α-syn pathology in the substantia nigra pas compacta (SNpc), low magnification (10x) confocal images of 10 sections/animal, collected every four, were counted using Fiji v2.0.0 software. For each brain slice, the α-syn pathology was expressed as the percentage of phospho α-syn positive TH neurons normalized to the total number of TH neurons in the ipsilateral side. For the double labeling with the phospho-S129 α-syn and the conformation-specific SynO2 antibodies as well as the staining with the rodent specific D37A6 antibody, sections were treated with Proteinase K (PK) (Sigma-Aldrich, USA) at a final concentration of 5 μg/ml in PBS for 10 min at RT to expose antigenic sites^27^. For the characterization of the pathological α-syn accumulations, sections were incubated with PK (5 μg/ml in PBS) at 37 °C for 1 h, in order to assess whether the inclusions were PK resistant^28^.

### Preparation of recombinant mouse α-syn monomer and α-syn PFFs

Recombinant mouse α-syn was produced in *E*. *coli* BL21 (DE3) transformed with the plasmid pD454-SR mouse α-syn (Addgene #89075) and expression of the recombinant protein was induced by addition of IPTG (1 mM). Cells were harvested by centrifugation (5000 x *g*, 4 °C), resuspended in lysis buffer (50 mM Tris-HCl, 50 mM KCl, 5 mM Mg(CH_3_COO)_2_, 10 mM EDTA, 0.3 M PMSF, pH 8.5), and lysed by three cycles of freeze-thaw. The lysate was centrifuged (14,000 x *g*, 4 °C) and the supernatant was boiled for 15 min and centrifuged (14,000 x *g*, 4 °C), then, loaded onto an ion exchange column (HiPrep Q FF 16/600, Cytiva) connected to an FPLC system (NGC Quest 10, Biorad). The column was washed with 20 mM Tris-HCl, pH 8.0, and was eluted by a 0–50% NaCl gradient. Protein-containing fractions were combined and loaded onto a size exclusion column (HiLoad Superdex 26/600 200 pg, Cytiva) and mouse α-syn was eluted in 50 mM Tris-HCl, 150 mM NaCl, pH 7.5. The purified protein was dialyzed against ultrapure water, lyophilized, and stored at −80 °C. Lyophilized mouse α-syn was reconstituted in PBS and diluted to 5 mg/ml. Fibrils were generated by incubation of the monomeric protein at 37 °C with orbital shaking (1000 x rpm) for 7 days. Aliquots of 20 μL were removed daily and analyzed to confirm fibrilization.

### Preparation of recombinant human monomeric α-syn and α-syn preformed fibrils (PFFs)

The plasmid pT7-7 α-syn WT (Addgene 36046)^33,47^ encoding for human α-syn was transformed into *E. coli* strain BL21 (DE3) (Sigma-Aldrich). Protein expression was induced in Luria-Bertani (LB) medium containing ampicillin (100 μg/ml) with isopropyl β-d-1-thiogalactopyranoside (IPTG, 1 mM) at an optical density (600 nm) of 0.4–0.7 and allowed to proceed at 37 °C for 3 h with shaking (250 x rpm). Subsequently, the cells were harvested by centrifugation (5000 × *g*; 4 °C), resuspended in lysis buffer (50 mM Tris, 50 mM KCl, 5 mM Mg(CH_3_COO)_2_, 10 mM EDTA, pH 8.5, 0.3 mM PMSF) and lysed by sonication followed by boiling for 20 min. Then, the lysate was centrifuged (10,000 × *g*; 4 °C) and the supernatant was collected. The α-syn was purified by ion exchange chromatography (IEX) followed by size exclusion chromatography (SEC). In brief, α-syn was loaded onto a HiPrep Q FF 16/10 anion exchange column (GE Healthcare Biosciences) on a fast protein liquid chromatography (FPLC) system (NGC Quest 10, Bio-Rad) and washed with 20 mM Tris-HCl, pH 8.0. α-syn was eluted against a linear gradient of 13% to 50% of 20 mM Tris-HCl, 1 M NaCl, pH 8.0. The collected fractions containing α-syn were pooled and loaded onto a HiLoadm 26/600 Superdex 200 prep grade column (GE Healthcare Biosciences) for further purification by SEC. α-syn was eluted in 50 mM Tris-HCl, 150 mM NaCl pH 8.0 and then, dialyzed overnight in ultrapure H_2_O, lyophilized, and stored at −80 °C. To generate α-syn PFFs the monomeric recombinant α-syn was diluted to 5 mg/ml in Phosphate Buffered Saline (PBS), vortexed, and then incubated at 37 °C with vigorous shaking at 1000x rpm for 7 days in an orbital thermomixer (Thermo Scientific), when formation of PFFs was verified by visual appearance, Thioflavin-T (ThT) assay, and sedimentation assay. Human PFFs (designated H2) generated by Dr Omar El-Agnaf (Qatar Biomedical Research Institute) were also used in some occasions to verify reproducibility of PFF cleavage.

### Thioflavin-T (ThT) fluorescence assay

At different time points during α-syn aggregation (5 mg/ml), 50 μl were withdrawn and mixed with PBS and ThT at a final concentration of 5 μΜ, then incubated in the dark for 10 min, and the fluorescence intensity was measured in 1 ml quartz fluorescence cuvette at 482 nm following excitation at 445 nm, using a Perkin-Elmer fluorescence spectrometer.

### α-syn sedimentation assay

The α-syn PFFs were diluted 1:10 in PBS and centrifuged for 30 min (10,000 x rpm, RT). The pellet was separated from the supernatant and resuspended in PBS. Equal volumes from both the supernatant and pellet fractions were analyzed by SDS-PAGE. Successful polymerization was demonstrated by the increased accumulation of α-syn in the pellet fraction compared to the supernatant on the final day of polymerization (*t* = 7 days).

### Proteomic analysis

For detection of EV-associated proteases, intact EVs were sonicated in PBS. Three biological samples with four technical replicates were used for the proteomic analysis where proteins were digested into peptides that were separated through nanoLC, ionized through nanoESI, and analyzed by a Q-Exactive Orbitrap HF-X spectrometer (Thermo Fisher Scientific). Specifically, the EV-associated proteins were digested according to the SP3 method using a mixture of trypsin and LysC (Promega) (Hughes et al., 2019). The generated peptidic samples were evaporated, solubilized in 0.1% formic acid in water, sonicated, and their peptide concentration was determined through UV NanoDrop measurement at A_280_.

An alternative proteomic sample preparation was used for the gel-separated α-synuclein in order to map internal cleavage sites that was based on the in-gel digestion protocol^48^.

Peptides (500 ng) were injected and pre-concentrated on a C18 trap column (Acclaim pepmap, Thermo Fisher Scientific) and then loaded onto a 50 cm C18 column (Acclaim pepmap, Thermo Fisher Scientific), operated at 60 °C. Binary pumps of the HPLC (RSLCnano, Thermo Scientific) consisted of solution A (100% H_2_O in 0.1% v/v formic acid) and solution B (100% ACN in 0.1% formic acid). Separation of the peptides was performed using a flow rate of 300 nl/min and gradual changes in the ratios between gradients A and B, with a total of 100 min long run. Full scan (MS1) spectra were acquired in the orbitrap using profile mode with a data-dependent acquisition (DDA) method.

### Protein identification and quantification

The generated mass spectral raw files were processed using MaxQuant software (Cox & Mann, 2008) (version 1.6.14.0). Trypsin was defined as the protease, and its specificity was set to full in the case EV cargo identification and to semi-specific in the case of mapping α-syn cleavage sites. Two missed cleavages were allowed for the database search. Minimum peptide length was set to 7 amino acids. Carbamidomethylation of cysteine was defined as static modification. Acetylation of the protein N-terminus, oxidation of methionine, and deamidation of asparagine and glutamine residues were set as variable modifications. False discovery rate (FDR) for both peptides and proteins was set to 1%. Label-free quantification (LFQ) was performed in order to calculate protein abundances. The “match between run” and “second peptides” options were enabled. The MS data were searched against a reviewed canonical FASTA database of *Mus musculus* from UniProt.

### Proteomic data analysis

Data visualization and statistical analysis of protein LFQ data generated from MaxQuant search engine were performed in the environment of Perseus program (Tyanova et al., 2016) (version 1.6.1.3). In both datasets, proteins were filtered for potential contaminants, reversed hit, and those that were only identified by site. Protein LFQ intensities were log2 transformed. The three biological and four technical replicates were used for our proteomic dataset. Biological samples were grouped together and filtered to obtain a minimum of 70% valid values in at least one group. The mass spectrometry proteomics data have been deposited to the ProteomeXchange Consortium *via* the PRIDE^49^ partner repository with the dataset identifier PXD044320.

### Enrichment analysis

Gene Ontology (GO) Enrichment analysis for biological processes was performed using DAVID functional annotation web tool^50,51^ (version 6.8) with official gene symbol as identifier, the *Mus musculus* as background, and the GOTERM_DIRECT annotation categories. A *p*-value of 0.05 was used as cut-off criterion. The top-20 processes were selected based on their *p*-values. Enrichment of proteins involved in biological pathways was performed using the KEGG pathway database. Top-20 pathways were selected based on their *p*-values and presented. Results were visualized in bar plots using Prism 8.0 software (GraphPad). Venn diagrams were created using the VENNY 2.1 online tool.

### Proteolytic processing of α-syn hPFFs by EVs and seeding capacity

Purified EVs were incubated with α-syn PFFs at a ratio of 1:2 w/w in PBS for 24 hours at 37 °C in the presence or absence of a cocktail of protease inhibitors containing AEBSF (1 mM), aprotinin (800 nM), E-64 (15 μΜ), EDTA (5 mM), and leupeptin (20 μΜ). These reactions (1 μΜ PFFs) were then incubated with monomeric α-syn (50 μΜ) in an orbital thermomixer (Thermo Scientific) over a 5-day period at 37 °C with shaking at 1000x rpm, and aggregation kinetics were monitored by ThT fluorescence assay.

### Enzymatic degradation of α-syn and inhibitor profiling

Aliquots of the purified EVs were incubated in 50 μl of PBS containing purified monomeric or α-syn PFFs at a ratio of 1:2 w/w (100 ng α-syn:200 ng EVs) for various times at 37 °C^34^. Reactions were terminated by the addition of SDS sample buffer and analyzed by Western blotting. For inhibition studies, the reactions were incubated for 16 h in the presence or absence of the following protease inhibitors: pepstatin A, 10 μΜ; EDTA, 1 mM; E-64, 4-10 μΜ; bestatin, 10 μM; marimastat, 10 μM; 1,10-phenanthroline, 10–100 μM; aprotinin, 10 μΜ; chymostatin, 100 μM; 8-hydroxy-5-nitroquinoline as an inhibitor for cathepsin B, 400 μM; LHVS, an irreversible, potent and selective inhibitor for cathepsin S at 100 nM. All inhibitors were purchased from Sigma-Aldrich, leupeptin, 100 μM (Roche); calpain inhibitor, 1 μM (Roche); MMP2 inhibitor III, 5 μM (Merck); and MMP9 inhibitor I, 5 μM (Merck). The Halt protease inhibitor cocktail (Thermo Fisher) was used as suggested by the manufacturer.

### In vitro proteolytic degradation of α-syn by cathepsin S

In total, 200 ng of in-house produced recombinant human α-syn were incubated with active recombinant human cathepsin S (Millipore #219343) in PBS pH 7.3 at 37 °C for 24 h. Then, the enzymatic reactions were terminated by addition of SDS-PAGE loading dye (10% SDS, 30% glycerol, 450 mM Tris-HCl pH 6.8, 0.12% bromophenol blue, 25% β-mercaptoethanol) and were analyzed by Western blotting using the Syn-1 antibody (BD Βiosciences, #610787) at 1:1000 dilution. The cathepsin S inhibitor LHVS (Sigma, SML2857) was added at 100 nM. It should be noted that at this concentration range, LHVS is a selective inhibitor of cathepsin S^52,53^. The enzymatic activity of recombinant cathepsin S was confirmed using the Z-Phe-Arg-AMC substrate (Sigma Aldrich, C9521) at a concentration of 10 nM (not shown).

### SDS-PAGE and western blotting

Following treatments, neuronal cells were collected in ice-cold PBS and centrifuged at 500 x *g* for 10 min at 4 °C. Pellets were lysed with lysis buffer (50 mM Tris-HCl pH 7.6, 150 mM NaCl, 1% Triton-X, 2 mM EDTA, and protease phosphatase inhibitors). Lysates were sonicated (five times for 0.5 sec, 20% amplitude and 0.8 cycle) at 4 °C, incubated for 30 min at 4 °C and centrifuged at 15,000 × *g* for 30 min at 4 °C. Pellets were further dissolved in 1% sarcosyl buffer, containing 10 mM Tris-HCl (pH 7.5), 10 mM EDTA, 150 mM NaCl, and protease-phosphatase inhibitors, and lysates were sonicated (five times for 2 s, 50% amplitude and 0.8 cycle). Protein concentrations were determined by the Bradford assay. Denaturing electrophoresis was performed on 13% SDS-PAGE. Lysates were mixed with 4xLaemmli buffer prior to running on the gels, then transferred onto nitrocellulose membranes (Whatman) and analyzed by Western blotting with antibodies specific against the target proteins. Primary antibodies are enlisted in Supplementary Table I (Supplementary Material). Subsequently, blots were probed with horseradish peroxidase-conjugated secondary antibodies, visualized with Clarity Max ECL Substrate (Bio-Rad), and exposed to Super RX film (Fuji).

### Protease activity assays

Proteolytic activities were measured using the synthetic fluorogenic substrate Z-Phe-Arg-AMC (Sigma-Aldrich). Intact EVs (4.5 μg) were added to assay buffer (PBS, pH 7.2–7.4) containing the substrate Ζ-Phe-Arg-AMC (75 μM). The following inhibitors of the different protease specificities were used: Ε-64 (10 μΜ), a general inhibitor of cysteine proteases, pepstatin A (10 μM), an inhibitor of aspartyl proteases, LHVS (100 nM), a selective cathepsin S inhibitor, and chymostatin (100 μΜ). Enzymatic activities were determined by monitoring the rate of increase in fluorescence emission at 440 nm following excitation at 370 nm with a Perkin-Elmer fluorescence spectrometer.

### Statistical analysis

Data are expressed as the mean ± standard error of the mean (SEM). The statistical analysis was performed with the GraphPad Prism 8 software (Version 8.2.0) (San Diego, CA), using non-parametric unpaired Mann-Whitney test (*N* < 6) for comparison between two groups.

## Supplementary information

Supplementary information

## Data Availability

The mass spectrometry proteomics data have been deposited to the ProteomeXchange Consortium via the PRIDE (Perez-Riverol et al.^49^) partner repository with the dataset identifier PXD044320.
