# Supplementary material for: Proteolytic activities of extracellular vesicles attenuate A-synuclein aggregation
Source: NPJ Parkinsons Dis. 2025 Sep 29;11:277. doi: 10.1038/s41531-025-01122-9 (PMC12480265; doi:10.1038/s41531-025-01122-9)
Supplement: Supplementary file 1 — Supplementary information [file 41531_2025_1122_MOESM1_ESM.pdf]

SUPPLEMENTARY INFORMATION

Table Supplementary I: List with antibodies used in total during the experiments.

Protein	Company	Cat. No.	Working dilution
**α-synuclein C-20 (human, mouse, rat)**	Santa Cruz<br>Rabbit polyclonal	sc-7011	WB 1:1000
**α-synuclein Syn-1 (human, mouse, rat)**	BD Biosciences<br>mouse monoclonal	610787	WB 1:1000
			IHC 1:2000
**α-synuclein MJFR-14 (MJFR)**	Abcam<br>rabbit monoclonal	138501	IF 1:500
**β-actin**	Cell Signaling<br>mouse monoclonal	4970	WB 1:1000
**γ-tubulin**	Sigma<br>mouse monoclonal	T5326	WB 1:1000
**β-tubulin III (Tuj-1)**	Sigma mouse monoclonal	T8578	IF 1:1000
**Flotillin-1**	Abcam mouse polyclonal	ab133497	WB 1:1000
**TSG101**	Invitrogen<br>monoclonal	4A10	WB 1:1000
**Goat anti-mouse IgG-HRP conjugated**	Biotium	20010	WB 1:7000
**Goat anti-rabbit IgG-HRP conjugated**	Biotium	20012	WB 1:7000
**Donkey anti-mouse Alexa 568**	Invitrogen	A10037	IF 1:2000
**Goat anti-mouse Alexa 488**	Biotium	20010	IF 1:2000
**Donkey anti-rabbit Alexa 568**	Invitrogen	1826664	IF 1:2000
**Goat anti-rabbit Alexa 488**	Invitrogen	1910795C	IF 1:2000

**α-synuclein D37A6 (mouse, rat)**	Cell Signaling rabbit monoclonal	4179	IF 1:2000
**α-synuclein SynO2**	BioLegend, mouse monoclonal	847602	IF:1000
**Anti Tyrosine Hydroxylase**	Merck Millipore mouse monoclonal	MAB 318	IF 1:2000
**Phosphorylated α-synuclein**	Abcam rabbit monoclonal	AB51235	IF 1:2000

Table Supplementary II. List of EV proteases identified in proteomic datasets.

Ctsd	Cathepsin D
Ctss	Cathepsin S
Ctsb	Cathepsin B
Blmh	Bleomycin hydrolase
Capn2	Calpain2
Capn5	Calpain 5
Uchl1	Ubiquitin C-Terminal Hydrolase L1
Uchl5	Ubiquitin C-Terminal Hydrolase L5
Usp5	Ubiquitin Specific Peptidase 5
Usp7	Ubiquitin Specific Peptidase 7
Usp9x	Ubiquitin Specific Peptidase 9 X-Linked
Usp14	Ubiquitin Specific Peptidase 14
Usp15	Ubiquitin Specific Peptidase 15
Otub1	OTU Deubiquitinase, Ubiquitin Aldehyde Binding
Pigk	Phosphatidylinositol Glycan Anchor Biosynthesis Class K, GPI-anchor transamidase
Lta4h	Leukotriene A4 Hydrolase
Trhde	Thyrotropin Releasing Hormone Degrading Enzyme

Lnpep Leucyl And Cystinyl Aminopeptidase

Rnpep Arginyl Aminopeptidase

Thop1 Thimet oligopeptidase

Nln Neurolysin

Adam9 Disintegrin and metalloproteinase domain-containing protein 9

Adam10 Disintegrin and metalloproteinase domain-containing protein 10

Adam11 Disintegrin and metalloproteinase domain-containing protein 11

Adam22 Disintegrin and metalloproteinase domain-containing protein 22

Adam23 Disintegrin and metalloproteinase domain-containing protein 23

Dpp3 Dipeptidyl peptidase 3

Cpe Carboxypeptidase E

Cpd Carboxypeptidase D

Ide Insulin Degrading Enzyme

Uqcrc2 Ubiquinol-Cytochrome C Reductase Core Protein 2

Uqcrc1 Ubiquinol-Cytochrome C Reductase Core Protein 1

Lap3 Cytosol aminopeptidase

Xpnpep1 X-Prolyl Aminopeptidase 1

Dnpep Aspartyl aminopeptidase

Cndp2 Cytosolic non-specific dipeptidase

Acy1 Aminoacylase 1

Folh1 Glutamate carboxypeptidase 2

Tfrc Transferrin Receptore

Ncln Nicalin, aspartyl protease

Crmp1 Collapsin Response Mediator Protein 1

Dpysl2 Dihydropyrimidinase Like 2

Dpysl3 Dihydropyrimidinase Like 3

Dpysl4 Dihydropyrimidinase Like 4

Dpysl5 Dihydropyrimidinase Like 5

Gda Guanine Deaminase

Psmd14 Proteasome 26S Subunit, Non-ATPase 14, deubiquitinase

Cops5 COP9 Signalosome Subunit 5

Cops6 COP9 Signalosome Subunit 6

Scrn1 Secernin 1

Scrn3 Secernin 3

Gpr56 Adhesion G Protein-Coupled Receptor G1, Mixed (C, S, T) catalytic type

Lphn2 Latrophilin 2 (Adhesion G Protein-Coupled Receptor L2) Mixed (C, S, T) catalytic type

Psma1 Proteasome 20S Subunit alpha 1

Psma2 Proteasome 20S Subunit alpha 2

Psma3 Proteasome 20S Subunit alpha 3

Psma4 Proteasome 20S Subunit alpha 4

Psma5 Proteasome 20S Subunit alpha 5

Psma6 Proteasome 20S Subunit alpha 6

Psmb1 Proteasome 20S Subunit beta 1,

Psmb2 Proteasome 20S Subunit Beta 2

Psmb3 Proteasome 20S Subunit Beta 3

Psmb5 Proteasome 20S subunit beta5

Psmb4 Proteasome 20S Subunit Beta 4

Psmb6 Proteasome 20S Subunit Beta 6,

Psmb7 Proteasome 20S Subunit Beta 7

Psmd7 Proteasome 26S Subunit, Non-ATPase 7 deubiquitinase

Asrgl1 Asparaginase And Isoaspartyl Peptidase 1

Gmps Guanine Monophosphate Synthase ligase, NA

Park7	Parkinsonism Associated Deglycase (DJ1)
Dag1	Dystroglycan 1,
Pcsk1	Proprotein Convertase Subtilisin/Kexin Type 1,
Pcsk2	Proprotein Convertase Subtilisin/Kexin Type 2,
Tpp2	Tripeptidyl-peptidase 2,
Prep	Prolyl Endopeptidase, short peptides
Apeh	Acylaminoacyl-Peptide Hydrolase
Prepl	Prolyl Endopeptidase Like

Table Supplementary III

**Protease Inhibitor**	**Family**
Aprotinin	Serine proteases
Bestatin	Serine Proteases
E-64	Amino peptidases, cysteine cathepsins
EDTA	Metalloproteases
Leupeptin	Serine and Cysteine proteases
Pepstatin A	Aspartic Acid Proteases
Marimastat	Matrix Metalloproteases (MMP)
phenanthroline	Matrix Metalloproteases (MMP)
chymostatin	chymotrypsin-like serine proteases, Cathepsin B
5-Nitro-8-hydroxyquinoline	Cathepsin B
LHVS	cysteine protease, Cathepsin S inhibitor
calpain inhibitor I	cathepsin B
MMP2 inhibitor III	MMP2
MMP9 inhibitor I	MMP9
epoxomycin	proteasome irreversible inhibitor
lactacystin	proteasome reversible inhibitor

Supplementary Figure 1

A.

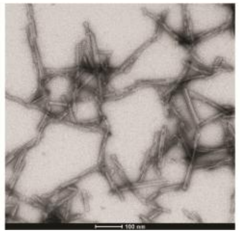

B.

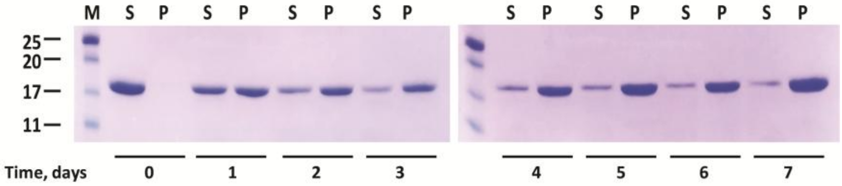

Supplementary Figure 2

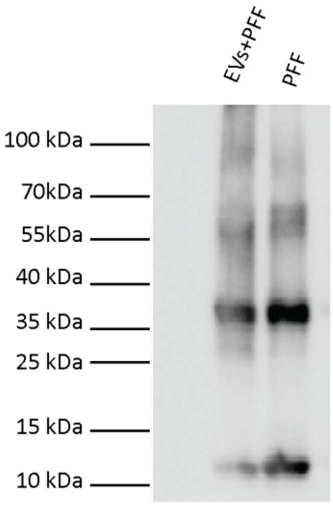

Supplementary Figure 3

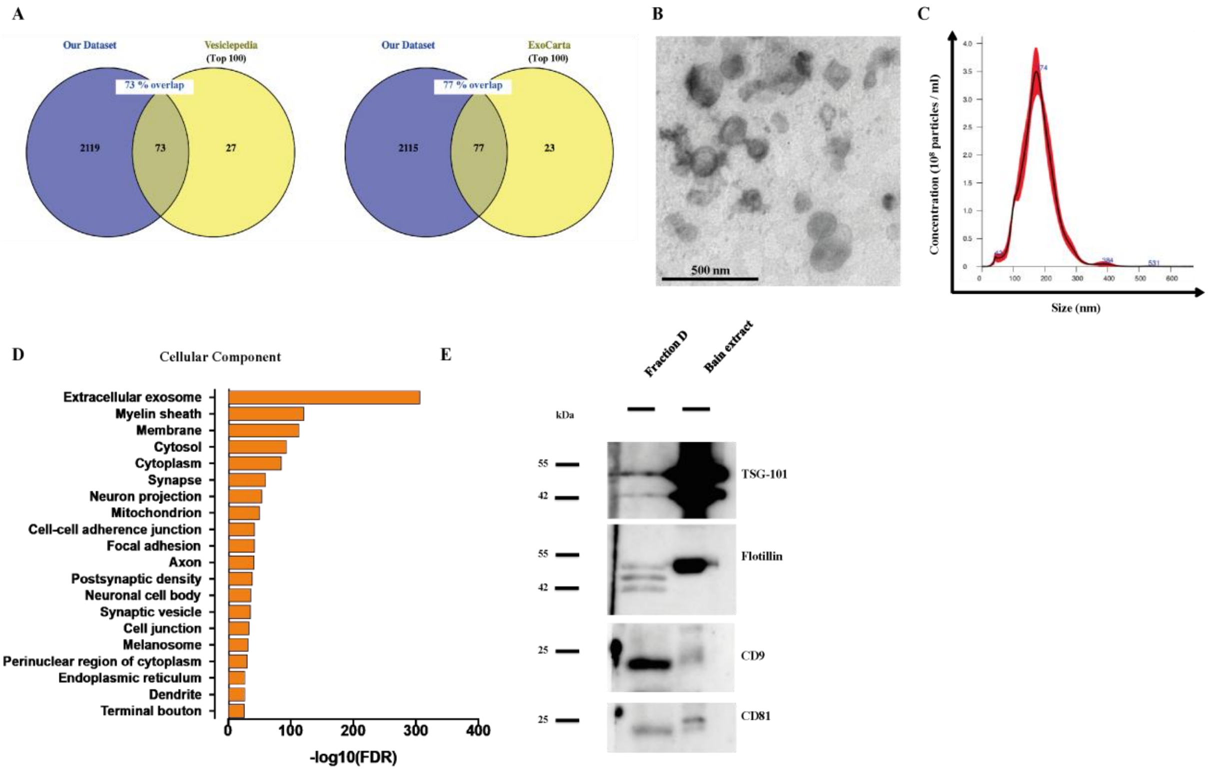

Supplementary Figure 4

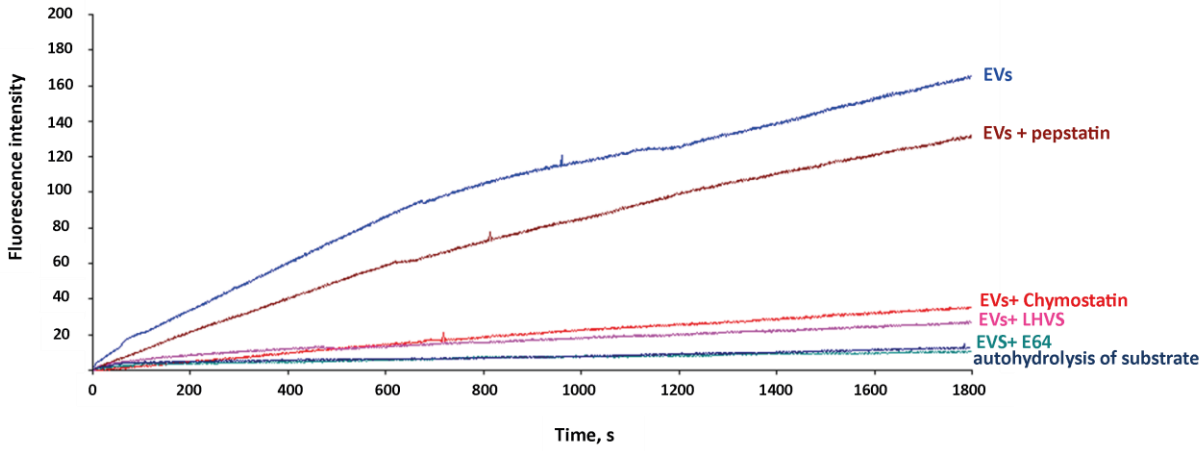

Supplementary Figure 5

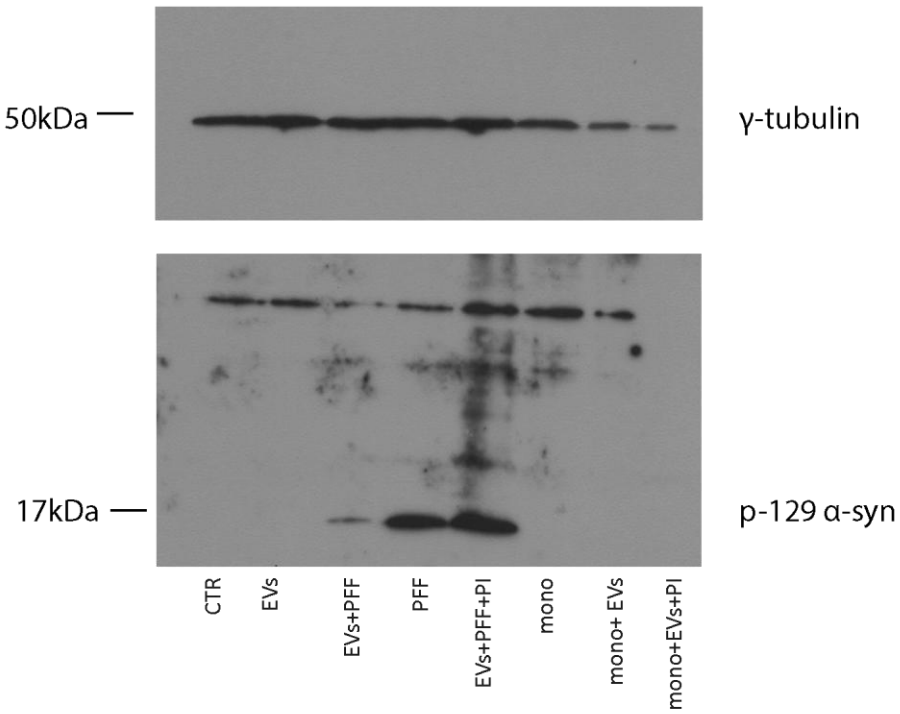

Supplementary Figure 6.

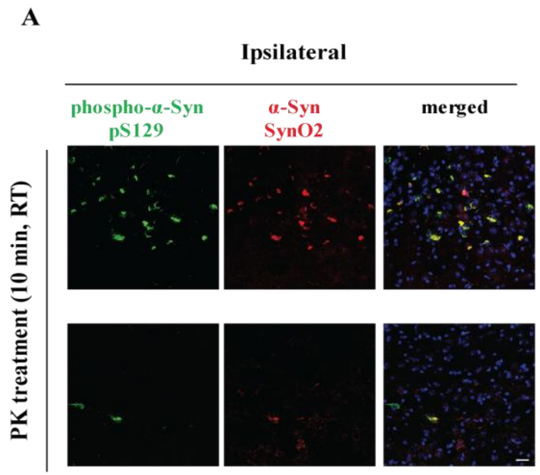

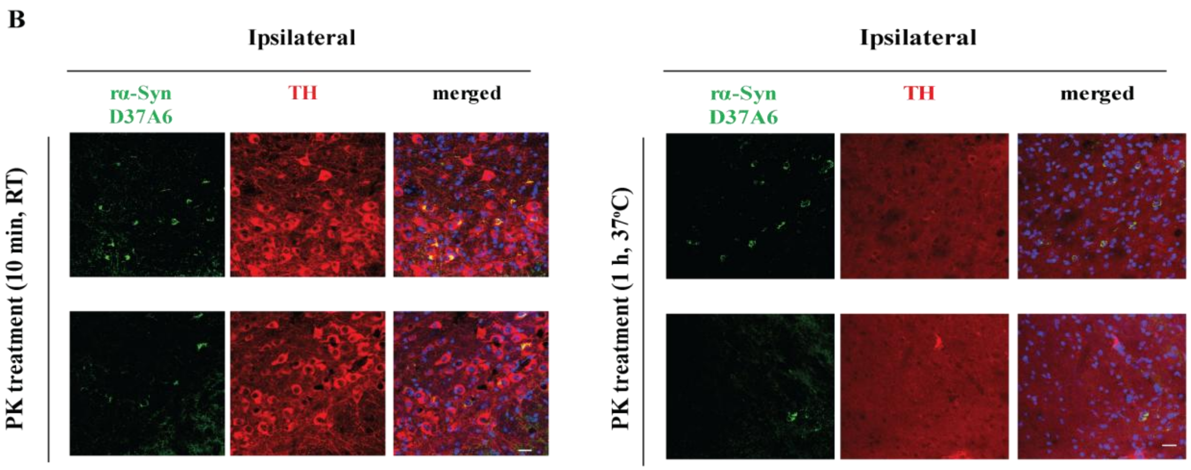

Supplementary Figure 7

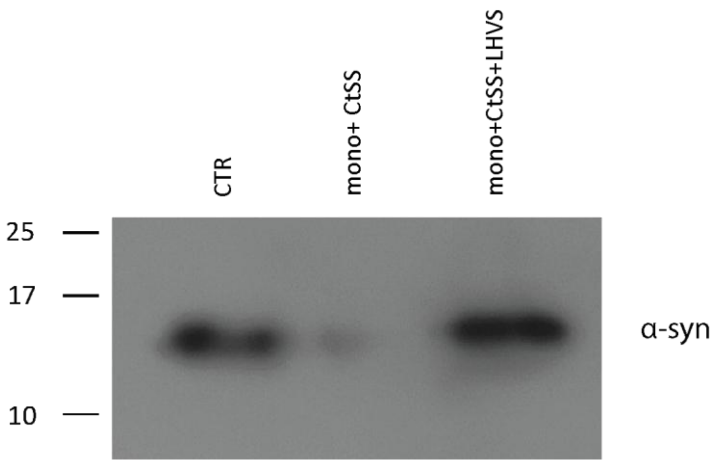

SUPPLEMENTARY FIGURE LEGENDS

Supplementary Figure 1. Characterization of α-syn PFFs. **(A)** Successful fibril formation was verified by centrifugation of PFFs and analysis by SDS-PAGE (15% polyacrylamide). Increasing amounts of α-syn appears in the pellet with progressing incubation times, indicating fibrilization of the soluble α-syn monomer. S: supernatant fraction, P: pellet fraction, M: molecular weight marker. **(B)** Representative EM image showing sonicated human PFFs.

Supplementary Figure 2. Conditioned medium-isolated EVs cleave hPFFs. EVs isolated from the conditioned medium of 48h cultured SHSY5Y cells were incubated with PFFs o/n at 37°C (EVs +PFF) at a ratio w/w of 2:1. α-syn levels were assessed using the C-20 antibody. hPFFs alone were used as control.

Supplementary Figure 3. Proteomic datasets of EVs are enriched in EV markers. (A) Venn diagrams showing the overlap between the proteins found in the proteomic dataset that originates from isolated EVs (purple) and the top 100 markers found in Vesiclepedia and ExoCarta databases (yellow). A total of 73 and 77 EV and EV markers, respectively, were found in our dataset. **(B)** Representative image EM image of isolated EVs showing enrichment in vesicles with EV characteristics. Scale bar, 500 nm. **C**. Representative NTA curve of the EV size distribution. **D** Graph depicting the top 20 hits in the cellular components analysis along the Y-axis and their statistical significance [-log(*P* value)], following analysis by DAVID platform. **E.** Representative WB analysis showing specific EV markers in the isolated brain EVs.

Supplementary Figure 4. Analysis of proteolytic activities of brain EV with kinetics. The activities of intact EVs were measured using the Z-Phe-Arg-AMC synthetic fluorogenic substrate in the presence or absence of the following inhibitors: E-64 (cysteine proteases) or pepstatin A (cathepsin D) or LHVS (cathepsin S) or chymostatin (cathepsin B). The increase in fluorescence corresponds to proteolysis rates and was monitored as described in Materials

and Methods. E-64 blocked the enzymatic activities completely, pepstatin A only partially, while strong inhibition is observed with chymostatin and LHVS.

Supplementary Figure 5. EV-induced proteolysis of human α-syn PFFs produces species that do not aid the phosphorylation of endogenous α-syn. Primary cortical neurons (5div) were treated for 72 hours with hPFFs, hPFFs pre-incubated with EVs (Exo) in the presence or absence of protease inhibitors (PIs), as well as EVs alone. Similar incubations were used with monomeric α-syn (mono). Untreated cells were used as control (CTR). (A) Immunoblotting for α-syn with the phospho-specific antibody to S-129 revealed that EV association with PFFs produces fragments that do not promote endogenous phospho-α-syn levels. γ-tubulin was used as loading control. (B)

Supplementary Figure 6. Mice inoculated with EV-cleaved PFFs exhibit reduced α-Syn pathologic accumulations, at 1 month post inoculation. (A) Representative high magnification confocal images of the ipsilateral SNpc of PFF and EV-Cleaved PFF-injected, 1-month post-injection, showing double immunostaining of phosphorylated-α-Syn (green) and conformation specific antibody SynO2 (red). (63x magnification, scale bar 20 μm). **(B)** Representative confocal images illustrating nigral accumulations for the rodent-specific α-Syn antibody (D37A6, green), following PK treatment at RT for 10 min and at 37oC for 1h, under which TH signal (red) is lost. (63x magnification, scale bar 20 μm).

Supplementary Figure 7. Cleavage of α-syn by cathepsin S. Active recombinant human cathepsin S (2.6 milliunits) was added to human monomeric α-syn substrate and incubated at 37°C for 24 hours. The resulting products were analyzed by Western blotting using the Syn-1 antibody specific for α-syn. In the absence of the enzyme, α-syn was unaltered (lane 1) but it was completely cleaved by cathepsin S (lane 2). Cleavage of α-syn was fully abolished by the inhibitor LHVS of cathepsin S added at 100 nM (lane 3). The amount of α-syn was 200 ng. Size markers are shown on the left in kDa.
